# Complete Genome Sequence of a Novel Human Betapapillomavirus Isolated from a Skin Sample

**DOI:** 10.1128/genomeA.01642-16

**Published:** 2017-03-30

**Authors:** Sankhadeep Dutta, Alexis Robitaille, Dana E. Rollison, Massimo Tommasino, Tarik Gheit

**Affiliations:** a Infections and Cancer Biology Group, International Agency for Research on Cancer, Lyon, France; b Department of Cancer Epidemiology, Moffitt Cancer Center, Tampa, Florida, USA

## Abstract

We report the genetic characterization of a new papillomavirus (HPV_MTS1) isolated and fully cloned from a skin swab. The L1 open reading frame of HPV_MTS1 was 85% identical to its closest human papillomavirus (HPV) type 80, which belongs to the species beta-2 of the genus *Betapapillomavirus*, hence qualifying it as a new HPV type.

## GENOME ANNOUNCEMENT

Human papillomaviruses (HPVs) that belong to the *Betapapillomavirus* genus (beta-HPVs) are broadly classified as cutaneotrophic viruses (1). More than 50 beta-HPV types have been identified to date and are widely prevalent in the skin of normal individuals (2, 3). However, this list continues to expand as many new beta-HPVs have recently been isolated from specimens of skin, oral cavities, and other anatomical sites (4, 5). Here, we report the complete genomic sequence of a novel HPV type obtained from the skin swab of a healthy individual.

The complete genome of a new HPV type (HPV_MTS1; 7,405 bp) was obtained by amplifying skin swab DNA using multiply primed rolling circle amplification (RCA) according to the manufacturer’s instructions (Illustra TempliPhi 100 amplification kit, GE Healthcare, Piscataway, NJ). The amplified product was digested with *EcoRI* and cloned into the pUC19 vector for sequencing using the primer-walking strategy (GATC Biotech, Germany) which covered nucleotides (nt) 842 to 6,935 (6,094 bp) of the viral genome. Furthermore, a long-range PCR was performed on the RCA product as a template using TaKaRa LA Taq DNA polymerase (TaKaRa Bio Inc.) and HPV_MTS1 specific primers (forward: 5′-CATATTTTGTACCTTTGTCGTC-3′ and reverse: 5′-GTAAAGTACCTTTAAAAGCGGA-3′) to obtain the remaining part of the genome. Ampliﬁcation was performed for 35 cycles at 94°C for 30 s, 56°C for 30 s, and 72°C for 5 min and cloned in pCR-XL-TOPO vector using the TOPO XL PCR cloning kit (Invitrogen, Carlsbad, CA) and sequenced. The sequence was checked using the proof reading Pfu DNA polymerase (Agilent Technologies, Santa Clara, CA, USA).

A novel HPV type shares less than 90% sequence similarity to the closest papillomavirus type in the L1 open reading frame (ORF) (1). Pairwise comparison of the L1 ORF of HPV_MTS1 demonstrated 85% nucleotide homology to its closest HPV type 80, which belongs to the genus *Betapapillomavirus*, species beta-2. The overall nucleotide homology between HPV_MTS1 and HPV80 was 89% with a G+C content of 39.8%. The genomic organization of this virus is typical of cutaneotrophic HPVs, containing five early (E1, E2, E4, E6, and E7) and two late (L1 and L2) genes but no E5 ORF. The long control region (LCR) is 384 bp and located between the L1 and E6 genes. LCR contains one polyadenylation site (AATAAA) for L1 and L2 transcripts, four consensus palindromic E2-binding sites (ACC-N_6_-GGT = 2 and slightly modified ACC-N_5_-GGT = 2) and two TATA Boxes (TATAAA) for the downstream early promoter. The two conserved zinc-binding domains of the viral E6 protein [CxxC(x)_29_CxxC and CxxC(x)_30_CxxC] are separated by 36 amino acids (6). The zinc-binding domain [CxxC(x)_29_CxxC] and the LxCxE motif for interaction with pRB and its related proteins are present in the E7 protein (6). The presence of ATP-binding site (GPPDTGKS) in the carboxy-terminal of the E1 protein confirms its ATP-dependent helicase activity. The E4 protein is encoded from an internal start codon of the E2 ORF and completely overlaps it.

To conclude, the genetic characterization of HPV_MTS1 expands the species composition of beta-2 papillomaviruses.

### Accession number(s).

The complete genome sequence of HPV_MTS1 is available in GenBank under the accession no. KY349817.
